# Anxiety and political action in times of the Covid-19 pandemic

**DOI:** 10.1177/00471178221149632

**Published:** 2023-01-16

**Authors:** Andreja Zevnik

**Affiliations:** University of Manchester

**Keywords:** anxiety, Covid-19, pandemic, political action, racial justice

## Abstract

Since the beginning of the global Covid-19 pandemic in the spring of 2020 countries across the world have implemented various measures to contain the virus. They have restricted public gatherings, mobility and congregation of people at homes and in public places. These restrictions however did not stop another chain of events – the global Black Lives Matter (BLM) protests. In the summer of 2020 people across the globe mobilised to protest the police killing of George Floyd. In the UK the protest for Black Lives took place in all major cities, but they also continued weekly in smaller communities by ‘taking the knee’. What interests me in this contribution is how anxieties experienced during the global pandemic contributed to the mobilisation of large-scale political actions for racial justice and how might we consider anxiety as a mobilising force in political space in times of global pandemic in particular in the context of anti-racist protests such as BLM. This forum contribution opens by considering how global pandemic aided conditions for political action for racial justice, before discussing the role of anxiety in political mobilising. Here I first detailed how anxiety is understood in Lacanian psychoanalysis before considering what it tells us about the BLM protests for racial justice and specifically the removal of the Colston statue during the Bristol protest on June 7 2020.

In response to the global Covid-19 pandemic in the spring of 2020 governments across the world implemented numerous restrictive measures to contain the virus. They prohibited public gatherings and limited mobility and congregation of people at homes and in public places. The everyday lives of most people became confined to the walls of their homes and contact with friends and family became increasingly mediated through digital platforms. This new way of living however was at least temporarily interrupted by another chain of events – the global Black Lives Matter (BLM) protests, which started in the summer of 2020 as a response to the police killing of George Floyd, an unarmed African-American man. The recording of the murder reached global audience through social media and led to numerous and sustained protests across the world. In the United Kingdom protests focused on racism and colonial legacies prevalent across the institutions and structures of the society, while in the United States political actions moved from claims about structural and institutional racism to calls for abolitionism that being of the police, housing-markets or capitalism.^1^ The intersection of the ‘two pandemics’^2^ – the Covid-19 pandemic and racism linked to the extra-juridical killing of black men and women – created a particular political environment whereby everyday citizens acknowledged racism and racial oppression and despite the restrictions began to organise.

As already noted in the introduction to this forum, the existing literature on the Covid-19 pandemic in international politics focuses on the pandemic’s political implications. Less, however, is said about the effects it had on individuals as political subjects. To date only medical sciences and psychologists considered how anxiety experienced during the pandemic changed peoples’ lives.^3^ However, in these works anxiety is used to describe a negative and out-of-ordinary experience, which needs to be ‘corrected’.^4^ It is primarily seen as a medical condition or a question of mental health. In contrast, my interest in this short contribution is how anxieties experienced during this global pandemic can be understood as facilitators of a productive political moment. How might anxiety work as a mobilising force in times of a global pandemic in particular when considered in the context of protests for racial justice?

If fear arises from an identifiable object, anxiety, according to Jacques Lacan is a disorienting reaction to something which cannot be located and yet it is there.^5^ The experience of anxiety is either immobilising and actions deriving from it reaffirm the familiar; or mobilising opening up possibility of different, transformative socio-political relations.^6^ Considering anxiety as a mobilising force departs from most literatures studying the socio-political effects of the phenomena, however the case of global BLM protests in the times of pandemic invites this consideration.^7^ Chua’s analysis of BLM protests in Minneapolis for example speaks to such mobilising potential. She states that ‘for Black youth . . . the riot expressed collective anguish [another word for anxiety] on both political and economic register, against a state order that . . . exchanged social wage for racialised violence and policing for plunder’.^8^ Similarly, Estellés with others demonstrates how discourses which presented youth as primary victims of the pandemic miss an important development in this groups’ political consciousness.^9^ They show how the everyday ‘boredom’ pushed young people in New Zealand ‘to reflect on their lives and fuelled their convictions to enact acts of citizenship’, these included virtual and physical support for BLM protests, LGBTQIIA+ rights and feminism.^10^

This contribution opens by considering how global pandemic might have created exceptional conditions for political action for racial justice, before discussing the role of anxiety in political mobilising. Here I will first detail how anxiety is understood in Lacanian psychoanalysis before considering what it tells us about the BLM protests for racial justice.

## Political action in times of the pandemic

While racial conflicts and police violence in the US and beyond has a long history, this pandemic created an opportunity in which protests for racial justice acquired a special political and moral resonance. Ètienne Balibar and Warren Montag note three distinct aspects of how the pandemic opened the possibilities of political action for racial justice. The first is what Balibar calls the ‘anthropological structure of the crisis’.^11^ Here, anthropological stands for a myriad of intersecting aspects which makes some groups more exposed and more vulnerable to the virus than others. This sanitary crisis, Balibar writes: ‘underlines and intensifies all sorts of inequalities, whether economic, urban, professional, or based on race and gender. [T]he virus is [. . .] more lethal for individuals with co-morbidities (which are socially determined), or living in conditions of poverty, or performing functions of care and domestic service for others’.^12^

Equally the measures which were put in place to control or suppress the pandemic do not protect everyone equally. As noted in *The Guardian*, while New York’s Governor Andrew Cuomo called the coronavirus a ‘great equaliser’, data shows the virus has been anything but indiscriminate.^13^ For example, reports from the Economic Policy Institute show that ‘African Americans face a higher risk of exposure to the virus, mostly on account of concentrating in urban areas and working in essential industries’.^14^ Only 20% of black workers reported being eligible to work from home compared with about 30% of their white counterparts.^15^ Some social groups remained more vulnerable to the virus due to their socio-economic status, living conditions and local geographies; while measures to protect against it only deepens already existing marginalisation and racialisation practices. The pandemic adds ‘new forms of discrimination to the already existing “structural” ones’.^16^ The pandemic thus transforms the multiple crisis (economic, social, health, racial) into a social condition in which these different intersecting aspects create new forms of social, economic and individual precarity and through means of social media heighten its visibility.

The second aspect concerns political consequences. The pandemic exposed failed governance reflected in the rise of right-wing populism and illiberal regimes supported by authoritarianism and authoritarian tendencies. The pandemic has exposed failed governance in matters of public health and other social services.^17^ This exposure ‘creates in the critical conjuncture of the pandemic a necessary condition of possibility for “federations” of protest movements against the system’.^18^ The movement for Black Lives becomes a testimony that movements can build federations which fight against devaluing of life, social security and for ‘a diferent kind of governance and authority’.^19^

Finally, the third aspect concerns the visibility of violence which in times of the pandemic took the form of unnecessary death. Warren Montag connects the ‘unnecessary death’ in the Covid-19 pandemic and in police violence with state inaction. The attempts to hide the numbers of deaths from the pandemic overdetermined the explosive reaction to the police killing of George Floyd and others.^20^ ‘The killing of unarmed African-Americans by police or white citizens with near impunity suddenly appeared as a pandemic of racist violence that, as in the case of the coronavirus, would be allowed to run its course’.^21^ However, it needs to be noted that state’s inaction is only inaction in as much as it does not attempt to prevent death. Its ability to kill indiscriminately is not unusual. It might have become more visible during the pandemic but the practice has a long history. As Silva notes, ‘in a country that is conditioned to carefully and systematically demarcate the value of human life based on numerous categories and hierarchies’ the indiscriminate killing is hardly a surprise.^22^

Balibar sees the effects of the pandemic to be distinctly cosmopolitan as it makes ‘our belonging as individuals to the same species a very material and perceptible phenomenon’.^23^ The three aspects – the anthropological structure of the crisis, its political consequences and the visibility of violence – are, I argue, also representations of the three moments of anxiety. Namely, the subject’s relation to authority, (racialised) belonging and its mobilising potential. The three aspects together create specific conditions under which we can begin to think transformative potential of this crisis and the impact it had on anti-racist organising in the summer of 2020 and beyond.

## Theorising anxiety

The most significant departure between the understandings of anxiety in every day and anxiety in psychoanalytic languages is that in psychoanalytic language anxiety is permanent. It is constitutive of modern political life and of liberal conceptions of subjectivity.^24^ This inherent bond between political subjects and anxiety does not mean that a subject lives in a state of permanent anxiety, frat or anguish. What it suggests, however, is that the social and political realities are constructed in a way that prevent the emergence of anxiety.^25^ The question to consider is then not whether or not anxiety is experienced, but about the difference in experiences. In other words, how well or how poorly social narratives (also known as fantasies) secure identities of different political subjects. For subjects whose individual identities overlap with fantasies, the experience of anxiety tends to be reduced to exceptional moments such as that of the pandemic; whereas for others whose identities depart from social fantasies (e.g. immigrants, those racialised as politically other), the experience of anxiety remains significantly more present.^26^ Following this logic, anxiety becomes productive of our everyday.

Theoretically the difference between subjectivities and identities emerges in the moment an individual becomes a political subject, in the moment one identifies or is recognised as belonging to a particular symbolic order, community, authority, nation.^27^ The emergence of political subject is bound with the intervention of language. One becomes a political subject in the moment when one becomes a speaking being.^28^ But in the moment of subject’s inception something is lost and the loss of this object determines subject’s political/social existence. Subject’s loss of completeness can be described in more political terms as the experience of alienation. Alenka Zupančič writes: ‘This fundamental loss or “alienation” is the condition of the thinking subject, the subject who has thoughts and representations. It is this loss that opens up the “objective reality” and allows the subject to conceive himself as a subject’.^29^ This foundational loss/alienation from its ‘primary’ existence also creates the conditions for anxiety not as an object driven experience but as a structural condition of subject’s existence. In other words, as political subjects we are always already anxious and will remain so. While subjects react to representation/images of anxiety, this representation tells us nothing about what triggered a reaction. Instead they tell us more about ‘the window of fantasy in the frame of which a certain object appears as terrifying’.^30^ Representations tell us more about what the subject imagines or creates as terrifying, it tells us about subjects’ vulnerability.

## Anxiety in times of the pandemic

Vulnerability at the heart of subject-formation is crucial for understanding political significance of anxiety. Political structures such as communities or states cannot exist with the ever present experience of danger and insecurity. They create fantasies to ensure relative safety and security for the majority of its citizens.^31^ However, the global pandemic which revealed the intersecting vulnerabilities of life, the growing inequalities between different communities and deepening racial, class, gender divides, dismantled these fantasies. It exposed the incompetence and failure of governance, in particular in relation to the management of life. The ‘fantasy of the social contract’ whereby the state has responsibility to provide good life and safety for its citizens took a particular blow, although as Silva reminds us, the obliviousness of states towards life should not surprise us.^32^ Pandemic only exposed the ever present vulnerability of life, it did not invent it.

There is a fundamental anxiety residing in the relationship between the state/authority and its political subject, which aims to cover the arbitrariness of this relation. The relationship is mediated by the fantasy of the social contract, which creates a particular subject, but also ascribes responsibilities to the state. When during the Covid-19 pandemic the fantasy was threatened the state attempted to patch it up by resorting to war-time analogies (Blitz spirit) or feel-good actions. In the UK one such gesture was weekly clapping for the staff working in the NHS (the National Health Services). This act of appreciation is also known as Clap for Our Carers.^33^

Anxiety emerges when there is too much or too little authority.^34^ The authority in BLM protests appears as both: too much in punitive killings of black men and women at the hands of the police, and too little in the form of excessive (also racialised) deaths due to the pandemic or states’ unwillingness to meaningfully engage in addressing racism and racial justice. Anxiety also triggers two different responses. It can mobilise for political change or in a regressive move it can return to the familiar symbols of order, power, state. For example, in the UK BLM protests in the summer of 2020 resulted in the tearing down of a statue of Edward Colston, a 17th century slave trader. The actions surrounding the Colston statue embody both dynamics. The global character of the protests speaks to shared experiences, be those of the failures of the state, the visibility of violence and injustice, or the character of the pandemic which through the intersecting crisis created new awareness of injustices. A coalition of protest groups, social movements and general public, I argue, create a fantasy that a different sharing of space and humanity is possible. The removal of the Colston statue on June 7 2020 represents a symbolic act of mobilisation for a different future.

However, the Colston statue was endowed with another symbolic value seen in the responses which condemned the acts and called for criminal charges: ‘In the eyes of the law a crime has been committed’.^35^ In these responses the Colston statue represents a symbol of colonial history, a continuation of the existing power relations, whose removal is seen as a direct attack on these values. Here Colston is an expression of anxiety which does away with the symbolic order that those invested in Colston defence know and benefit from.

However, even within the logic of anxiety which mobilises for political change a sense of ‘old and familiar’ can remain. The problem with anxiety is that it is not representable, and yet the subject can only experience that which achieves some form of representation. Zupančič introduces the idea of the sublime to highlight this trap.^36^ Sublime, for Zupančič, is a feeling which combines our insignificance ‘as far as the whole of the universe is concerned’ and the fact that what functions at the centre of ‘our ordinary life suddenly strikes us as trivial and unimportant’.^37^ It is the experience whereby one watches the most horrific disaster from a position of safety and experiencing ‘narcissistic satisfaction that emerges with the feeling of the sublime’.^38^ The subject observes a disaster from a position of detachment (alienation) and converts a feeling of anxiety into a certain gain (a pleasure). An attainment of pleasure from revolutionary acts is what Lacan sees as problematic.^39^ For Lacan a revolutionary progressive stance can only be achieved by hollowing enjoyment from political action. That is by not attaching political demands to that which is known, familiar or comforting. For example, not only policies for racial equality or a relatively peaceful removal of statues but actions which do away with structural racism, whiteness of socio-political structures and so on. Each political struggle will have demands which harvest on enjoyment, and others which are buried in anxiety and discomfort. The crucial moment in anti-racist political organising in anxious times is thus the insistence on discomfort of not knowing what change might bring.

Drawing on the idea of anxiety this contribution showed how pandemic has opened up a space for coalition-building and political action which is centred around shared experiences of vulnerability. The pandemic did not trigger new kinds of vulnerabilities about life, instead it exposed and opened up those already existing but perhaps less widely acknowledged. The challenge of political organising in time of the pandemic is then how to maintain the momentum of openness. The answer might lie in the insistence on anxiety, that is in the rejection of fantasies which offer a false sense of security for some at the expense of others.

## Notes

1. Charmaine Chua, ‘Abolition is a Constant Struggle: Five Lessons From Minneapolis’, *Theory & Event*, 23(4 Suppl.), 2020, pp. 128–9.

2. Kumarini Silva, ‘Covid-19 and the Mundane Practices of Privilege’, *Cultural Studies*, 35(2–3), 2021, p. 239.

3. Darren Courtney, Priya Watson, Marco Battaglia, et al., ‘COVID-19 Impacts on Child and Youth Anxiety and Depression: Challenges and Opportunities’, *The Canadian Journal of Psychiatry*, 65(10), 2020, pp. 688–91; Susanna Every-Palmer, Matthew Jenkins, Phillip Gendall, et al., ‘Psychological Distress, Anxiety, Family Violence, Suicidality, and Wellbeing in New Zealand During the COVID-19 Lockdown: A Cross-Sectional Study’, *PLoS One*, 15(11), 2020, e0241658. DOI: 10.1371/journal.pone.0241658.

4. Silva, ‘Covid-19 and the Mundane Practices of Privilege’, p. 243.

5. Jacques Lacan, *The Seminar of Jacques Lacan Book X: Anxiety* (Cambridge: Polity Press, 2014).

6. Andreja Zevnik, ‘Anxiety, Subjectivity and the Possibility of Emancipatory Politics’, *Journal of International Relations and Development*, 24(4), 2021, pp. 1050–6.

7. For more on this see special forum on anxiety, Jelena Subotić and Filip Ejdus (eds), ‘Forum: On Anxiety’, *Journal of International Relations and Development*, 24(4), 2022, pp. 1014–63.

8. Chua, ‘Abolition is a Constant Struggle’, p. 127.

9. Marta Estellés, Holly Bodman and Carol Mutch, ‘Beyond Victims and Villains: Young People’s Acts of Citizenship During Covid-19’, *Citizenship, Social and Economic Education*, 21(2), 2022, pp. 79–96.

10. Estellés et al., ‘Beyond Victims and Villains’, p. 11.

11. Etienne Balibar, ‘Living, Learning, Imagining in the Middle of the Crisis’, *Crisis and Critique*, 7(3), 2021, p. 15.

12. Balibar, ‘Living, Learning, Imagining’, p. 15.

13. Kenya Evelyn, ‘‘It’s a Racial Justice Issue’: Black Americans are Dying in Greater Numbers From Covid-19’, *The Guardian*, 8 April 2020, available at: https://www.theguardian.com/world/2020/apr/08/its-a-racial-justice-issue-black-americans-are-dying-in-greater-numbers-from-covid-19 (accessed 15 April 2022).

14. Evelyn, ‘It’s a Racial Justice Issue’.

15. Silva, ‘Covid 19 and the Mundane Practices of Privilege’, p. 242.

16. Balibar, ‘Living, Learning, Imagining’, p. 15.

17. Balibar, ‘Living, Learning, Imagining’, p. 16.

18. Balibar, ‘Living, Learning, Imagining’, p. 16.

19. Balibar, ‘Living, Learning, Imagining’, p. 16.

20. Warren Montag, ‘Acceptable Deaths: Killing and Letting Die in the Covid-19 Conjuncture’, *Crisis and Critique*, 7(3), 2021, p. 247.

21. Montag, ‘Acceptable Deaths’, p. 247.

22. Silva, ‘Covid 19 and the Mundane Practices of Privilege’, p. 241.

23. Balibar, ‘Living, Learning, Imagining’, p. 24.

24. John Cash, ‘Psychoanalysis, Cultures of Anarchy, and Ontological Security’, *International Theory*, 12, 2020, pp. 306–21; Charlotte Epstein, ‘Surveillance, Privacy and the Making of the Modern Subject: Habeas What Kind of Corpus?’, *Body and Society*, 22(2), 2016, pp. 28–57.

25. See: Zevnik, ‘Anxiety, Subjectivity and the Possibility of Emancipatory Politics’, pp. 1050–6; Alenka Zupančič, ‘The Subject of the Law’, in Slavoj Žižek (ed.), *Cogito and the Unconscious* (Durham and London: Duke University Press, 1998), pp. 41–72; Slavoj Žižek, *Sublime Object of Ideology* (London: Verso, 1992).

26. Ari Hirvonen, ‘Fear and Anxiety: The Nationalist and Racist Politics of Fantasy’, *Law & Critique*, 28(3), 2017, pp. 249–65; Zevnik, ‘Anxiety, Subjectivity and the Possibility of Emancipatory Politics’, pp. 1050–6.

27. Andreja Zevnik, ‘Postracial Society as Social Fantasy: Black Communities Trapped Between Racism and a Struggle for Political Recognition’, *Political Psychology*, 38(4), 2017, p. 629.

28. Žižek, *Sublime Object of Ideology*, p. 111.

29. Zupančič, ‘The Subject of the Law’, p. 66.

30. Zupančič, ‘The Subject of the Law’, p. 67.

31. Badredine Arfi, ‘Fantasy in the Discourse of ‘Social Theory of International Politics’’, *Cooperation and Conflict*, 45(4), 2010, pp. 428–48; Moran Mandelbaum, *The Nation/State Fantasy: A Psychoanalytical Genealogy of Nationalism* (London: Palgrave Macmillan, 2020).

32. Kumarini, ‘Covid 19 and the Mundane Practices of Privilege’, p. 242.

33. Justine Parkinson, ‘Coronavirus: At-Home Civil Servants ‘Showing Blitz Spirit’’, *BBC News*, 5 August 2020, available at: https://www.bbc.co.uk/news/uk-politics-53660963 (accessed 1 May 2021); Matt Hancock, ‘We Must all do Everything in our Power to Protect Lives’, *The Telegraph*, 14 March 2020, available at: https://www.telegraph.co.uk/politics/2020/03/14/must-do-everything-power-protect-lives (accessed 30 April 2021).

34. Jaco Barnard-Naudé, ‘‘She Reigns and he Does Not Govern’: The Discourse of the Anxious Hysteric in Post-Apartheid South Africa’, *Law & Critique*, 28(3), 2017, pp. 267–87; Zevnik, ‘Anxiety, Subjectivity and the Possibility of Emancipatory Politics’, pp. 1050–6.

35. Lara Choksey, ‘Colston Falling’, *Journal of Historical Geography*, 74, 2021, p. 82.

36. Alenka Zupančič, *The Ethics of the Real: Kant and Lacan* (London: Verso, 2011).

37. Zupančič, *The Ethics of the Real*, p. 152.

38. Zupančič, *The Ethics of the Real*, p. 153.

39. Jacques Lacan, *Seminar XVII: The Other Side of Psychoanalysis* (New York, NY: W.W. Norton, 2007).

